# Targeting the β_2_‐adrenergic receptor increases chemosensitivity in multiple myeloma by induction of apoptosis and modulating cancer cell metabolism

**DOI:** 10.1002/path.6020

**Published:** 2022-11-22

**Authors:** Hatice Satilmis, Emma Verheye, Philip Vlummens, Inge Oudaert, Niels Vandewalle, Rong Fan, Jennifer M Knight, Nathan De Beule, Gamze Ates, Ann Massie, Jerome Moreaux, Anke Maes, Elke De Bruyne, Karin Vanderkerken, Eline Menu, Erica K Sloan, Kim De Veirman

**Affiliations:** ^1^ Department of Hematology and Immunology, Myeloma Center Brussels Vrije Universiteit Brussel Brussels Belgium; ^2^ Laboratory of Myeloid Cell Immunology VIB Center for Inflammation Research Brussels Belgium; ^3^ Laboratory of Cellular and Molecular Immunology Vrije Universiteit Brussel Brussels Belgium; ^4^ Department of Clinical Hematology Universitair Ziekenhuis Gent Ghent Belgium; ^5^ Departments of Psychiatry, Medicine, and Microbiology & Immunology Medical College of Wisconsin Milwaukee WI USA; ^6^ Department of Clinical Hematology Universitair Ziekenhuis Brussel, Vrije Universiteit Brussel Brussels Belgium; ^7^ Neuro‐Aging & Viro‐Immunotherapy, Center for Neurosciences Vrije Universiteit Brussel Brussels Belgium; ^8^ Institute of Human Genetics, CNRS University of Montpellier Montpellier France; ^9^ Laboratory for Monitoring Innovative Therapies, Department of Biological Hematology CHU Montpellier Montpellier France; ^10^ Institut Universitaire de France Paris France; ^11^ Monash Institute of Pharmaceutical Sciences, Drug Discovery Biology Theme Monash University Parkville VIC Australia

**Keywords:** multiple myeloma, β‐blocker, propranolol, autophagy, metabolism, glucose, chemosensitivity, combination therapy

## Abstract

While multi‐drug combinations and continuous treatment have become standard for multiple myeloma, the disease remains incurable. Repurposing drugs that are currently used for other indications could provide a novel approach to improve the therapeutic efficacy of standard multiple myeloma treatments. Here, we assessed the anti‐tumor effects of cardiac drugs called β‐blockers as a single agent and in combination with commonly used anti‐myeloma therapies. Expression of the β_2_‐adrenergic receptor correlated with poor survival outcomes in patients with multiple myeloma. Targeting the β_2_‐adrenergic receptor (β_2_AR) using either selective or non‐selective β‐blockers reduced multiple myeloma cell viability, and induced apoptosis and autophagy. Blockade of the β_2_AR modulated cancer cell metabolism by reducing the mitochondrial respiration as well as the glycolytic activity. These effects were not observed by blockade of β_1_‐adrenergic receptors. Combining β_2_AR blockade with the chemotherapy drug melphalan or the proteasome inhibitor bortezomib significantly increased apoptosis in multiple myeloma cells. These data identify the therapeutic potential of β_2_AR‐blockers as a complementary or additive approach in multiple myeloma treatment and support the future clinical evaluation of non‐selective β‐blockers in a randomized controlled trial. © 2022 The Authors. *The Journal of Pathology* published by John Wiley & Sons Ltd on behalf of The Pathological Society of Great Britain and Ireland.

## Introduction

Multiple myeloma is an incurable plasma cell malignancy that develops in the bone marrow and eventually causes painful osteolytic lesions, cytopenia, anemia, and renal failure. The median age of diagnosis is approximately 66–70 years, with 37% of the patients younger than 65 years. Although the introduction of new treatment options has significantly prolonged the survival of multiple myeloma patients (5–7 years), long‐term treatment in combination with multi‐drug regimens has significantly increased the cost of treatment [1, 2]. Drug repurposing could therefore provide an alternative to supplement traditional chemotherapy approaches. As beta‐1 adrenergic receptor (β_1_AR) blockers are dominantly used in the clinic to treat cardiovascular diseases, we aimed to assess the therapeutic benefit of targeting either the β_1_AR, the beta‐2 adrenergic receptor (β_2_AR), or both βARs as a therapeutic option for multiple myeloma patients.

β‐Blockers block signaling from the sympathetic nervous system through blockade of adrenergic receptor (AR) subtypes, namely β_1_ (commonly associated with the heart), β_2_ (associated with vascular and airway relaxation), and β_3_ (present in the cells of brown adipose tissue from rats) [3]. Both *in vitro* and *in vivo* studies demonstrated that blockade of βAR signaling pathways could inhibit multiple processes associated with tumor progression including cell proliferation, invasion, migration, angiogenesis, and tumor immune response [4, 5, 6, 7]. Recently, a number of clinical observational studies found that β‐blocker therapy was associated with improved prognosis in breast [8], prostate [9], colorectal [10], ovarian [11], lung [12], and skin cancer [13] patients. A retrospective analysis of 1,971 newly diagnosed multiple myeloma patients indicated that β‐blocker use was associated with a reduced risk of disease‐specific death and overall mortality in comparison to non‐use of a β‐blocker [14, 15]. Moreover, a prospective study by Knight *et al* demonstrated that the β‐blocker propranolol regulated the bone marrow niche and changed the gene expression profile in favor of a better engraftment for hematopoietic cell transplant recipients [16]. Taken together, these data raise the possibility that β‐blockers may improve the treatment outcome of multiple myeloma patients.

Despite promising clinical data, the anti‐myeloma potential of βAR‐blockers and the βAR subtypes associated with the anti‐cancer effects of β‐blockers remain to be elucidated. In this study, we examined the impact of βAR subtype expression on overall survival in multiple myeloma patients. The anti‐tumor effect of both selective and non‐selective β‐blockers in multiple myeloma was investigated, as well as the effects on downstream signaling pathways and mechanisms including autophagy, apoptosis, glycolysis, and metabolic respiration. In addition, the β‐blocker propranolol was combined with standard‐of‐care agents to assess their potential to improve anti‐tumor effects in multiple myeloma.

## Materials and methods

### Gene expression data

The Total Therapy 2 (TT2) cohort contains microarray‐based gene expression data from 345 multiple myeloma patients from the University of Arkansas for Medical Sciences (UAMS, Little Rock, AR, USA; accession number GSE2658). Gene expression of *ADRB1*, *ADRB2*, *ADRB3*, hexokinase‐II (*HK2*), and glucose transporter 1 (*GLUT1*) was analyzed in bone marrow plasma cells (BMPCs) of healthy individuals (*n* = 22) and in patient samples at different multiple myeloma substages including monoclonal gammopathy of undetermined clinical significance (MGUS) (*n* = 44), smoldering multiple myeloma (SMM) (*n* = 12), and in CD138^+^ cells of newly diagnosed MM patients (*n* = 345). Gene expression of *ADRB1*, *ADRB2*, and *ADRB3* was analyzed in human MM cell lines using the Lombardi and Heidelberg‐Montpellier (HM) cohorts. These cohorts contain gene expression data of human MM cell lines (both *n* = 23). Gene expression data were normalized using the MAS5 algorithm and processing of the data was performed using the webtool GenomicScape. The prognostic value of *ADRB1*, *ADRB2*, and *ADRB3* expression for overall survival and progression‐free survival was evaluated in RNA sequencing data from the Multiple Myeloma Research Foundation (MMRF) CoMMpass Study (NCT01454297; started in 2011), which is a prospective study of newly diagnosed multiple myeloma patients.

### Patient samples and multiple myeloma cell purification

Bone marrow samples were collected for routine diagnostic or evaluation purposes after patients’ written informed consent was obtained and in accordance with the Declaration of Helsinki and institutional research board approval from Brussels University Hospital (B.U.N. 143201838414). First, mononuclear cells were isolated by density gradient centrifugation with Lymphoprep™ (STEMCELL™ Technologies, Grenoble, France). Then CD138^+^ cells were obtained by magnetic activated cell sorting (MACS) using human CD138 MicroBeads (Miltenyi Biotec, Gladbach, Germany) following the manufacturer's instructions.

### Drugs and reagents

Bisoprolol and propranolol were purchased from Selleckchem (Planegg, Germany). ICI‐118,551 was obtained from Sigma‐Aldrich (St Louis, MO, USA). Dimethyl sulfoxide (DMSO), obtained from MP Biomedicals (Illkirch Graffenstaden, France), was used as the solvent for bisoprolol and propranolol, whereas ICI‐118,551 was dissolved in sterile H_2_O. Bortezomib was purchased from Selleckchem and melphalan from Sigma‐Aldrich; both were dissolved in DMSO following the manufacturer's instructions. 3‐Methyladenine (3MA), obtained from Selleckchem, was dissolved in sterile H_2_O following the manufacturer's instructions.

### Cell lines and culture

Human multiple myeloma cell lines LP‐1, OPM‐2, RPMI‐8226, and ANBL‐6 cells, and the human stromal cell line HS‐5, were obtained from the American Type Culture Collection (ATCC, Molsheim, France). The XG‐2 cell line was kindly provided by J Moreaux (University of Montpellier, Montpellier, France). Myeloma cell lines and stromal cells were cultured in RPMI‐1640 and DMEM medium (Lonza, Basel, Switzerland) respectively, supplemented with 2 mm l‐glutamine (Thermo Fisher Scientific, Waltham, MA, USA), 1% penicillin/streptomycin stock solution (Thermo Fisher Scientific), and 10% fetal bovine serum (FBS; Hyclone, Logan, UT, USA) at 37 °C in 5% CO_2_. ANBL‐6 and XG‐2 cells were supplemented with 2 ng/ml recombinant IL‐6 (R&D Systems, Abingdon, UK). The authenticity of the human multiple myeloma cell lines was regularly confirmed by short tandem repeat analysis. The cell lines were checked monthly for mycoplasma contamination using the MycoAlert Mycoplasma Detection Kit (Lonza).

### RT‐qPCR

RNA was extracted by the RNeasy Mini Kit (Qiagen, Valencia, CA, USA) and 1 μg of RNA was converted into cDNA using a first‐strand cDNA synthesis kit (Thermo Fisher Scientific) in a Swift MiniPro Thermal Cycler (Esco LifeSciences, Changi, Singapore). Primer sequences were as follows: *ADRB1*: forward (5'‐CTG AGG GAT TTC TAC CTC ACA C‐3') and reverse (5'‐GCC TGG TCC TTC CAA CTA AT‐3'); *ADRB2*: forward (5'‐GAG CCT GCT GAC CAA GAA TAA‐3') and reverse (5'‐GAA TGG GCA AGA AGG TAA‐3'). *ADRB1* and *ADRB2* primers were obtained from Integrated DNA Technologies (Leuven, Belgium). SYBR Green (Applied Biosystems, Thermo Fisher Scientific) was used to perform quantitative real‐time PCR. Expression levels of mRNA were determined using the QuantStudio 12K Flex Real‐Time PCR System (Thermo Fisher Scientific). Each sample was amplified in triplicate. Transcripts of *GAPDH* (Hs_GAPDH_1_SG Quantitect Primer Assay, Qiagen) were measured for normalization.

### Cell viability and apoptosis assays

For viability assays, 20,000 CD138^+^ cells were seeded in a 96‐well plate and treated with β‐blockers for 24 h in 2% FBS supplemented medium. Human multiple myeloma cell lines and HS‐5 cells were subjected to β‐blocker treatment for 24 h in serum‐free medium. Cell viability analyses were performed using the CellTiter‐Glo Luminescent Cell Viability Assay (Promega, Madison, WI, USA). Luminescence was measured in opaque‐walled 96‐well plates using a GloMax luminometer (Promega).

For apoptosis analysis, human multiple myeloma cell lines were seeded in 24‐well plates at 150,000 cells/ml. For combination experiments with the autophagy inhibitor 3MA, cells were pretreated with 2 mm for 4 h; later, propranolol was added to the cells.

For combination experiments with standard‐of‐care agents, cells were pretreated with 10, 25, or 35 μm propranolol for 3 days. On the third day, 2, 3, or 4 nm bortezomib or 0.5, 1, or 2 μm melphalan was added to the cells. For apoptosis analysis, samples were stained with allophycocyanin (APC)‐coupled annexin V staining and 7‐aminoactinomycin D (7‐AAD) and analyzed by a FACSCanto flow cytometer (BD Biosciences, Erembodegem, Belgium).

### Western blotting

LP‐1 and XG‐2 cells were seeded in six‐well plates at 500,000 cells/ml and treated with propranolol for 24 h in serum‐free medium. After treatment, samples were collected and pellets were lysed in lysis buffer containing 50 mm Tris, 150 mm NaCl, 1% Nonidet P40, and 0.25% sodium deoxycholate. The following protease and phosphatase inhibitors were added: 4 mm Na_3_VO_4_, 1 mm Na_4_P_2_O_7_, 2 μg/ml aprotinin, 50 μg/ml leupeptin, 500 μg/ml trypsin inhibitor, 10 μm benzamidine, 2.5 mm *para*‐nitrophenyl benzoate (all from Sigma‐Aldrich); 50 mm NaF, 5 mm ethylenediaminetetraacetic acid (both from VWR International, Radnor, PA, USA); and 1 mm 4‐(2‐aminoethyl)benzenesulfonyl fluoride hydrochloride and 50 μg/ml pepstatin A (both from ICN Biomedicals, Costa Mesa, CA, USA). The protein content of the cell lysates was measured using a Pierce BCA (bicinchoninic acid) Protein Assay Kit (Thermo Fisher Scientific). Western blotting of cell extracts was performed as described previously [17]. The antibodies used for analysis were β_1_AR (#12271, 1/500), β_2_AR (D6H2, #8513, 1/1,000), BAX (#2772, 1/1,000), BAD (11E3, #9268, 1/1,000), BCL‐2 (50E3, #2870, 1/1,000), caspase‐3 (8G10, #9665, 1/1,000), caspase‐8 (1C12, #9746, 1/1,000), caspase‐9 (#9502, 1/1,000), Beclin‐1 (#3738, 1/1,000), SQSTM1/p62 (#5114, 1/500), microtubule‐associated protein 1A/1B‐light chain 3 (LC3‐I/II) (#4108, 1/1,000), hexokinase‐I (HK1) (C35C4, #2024, 1/1,000), hexokinase‐II (HK2) (C64G5, #2867, 1/1,000), LDHA (C4B5, #3582, 1/1,000), PKM2 (D78A4, #4053, 1/1,000), mTOR (7C10, #2983, 1/1,000), p‐mTOR (Ser2448, D9C2, #5536, 1/1,000), STAT3 (79D7, #4904, 1/1,000), p‐STAT3 (Tyr705, 3E2, #9138, 1/1,000), GLUT1 (#377282, 1/250), HIF‐1α (C‐term, #10006421, 1/500), β‐actin (#4967, 1/1,000), α‐tubulin (#2144, 1/1,000), horseradish peroxidase (HRP)‐coupled anti‐rabbit IgG (#7074, 1/2,500), and anti‐mouse IgG (#7076, 1/3,000). All of the antibodies were purchased from Cell Signaling Technology (Leiden, The Netherlands) except GLUT1, which was obtained from Santa Cruz Biotechnology (Heidelberg, Germany), and HIF‐1α, which was obtained from Cayman Chemicals (Ann Arbor, MI, USA). β‐Actin or α‐tubulin was used as a loading control. Chemiluminescence was analyzed using an Odyssey Fc Imager (LI‐COR Biosciences, Bad Homburg, Germany). The pixel densities of proteins were quantified using ImageJ, version 1.53 (https://imagej.nih.gov/ [last accessed 23 September 2022]).

### Mitochondrial oxygen flux and extracellular acidification analyses

*In vitro* cell metabolic alterations were measured using a Seahorse XFe96 Analyser (Agilent Technologies, Santa Clara, CA, USA). In brief, the day prior to assay, LP‐1 cells were plated in a 24‐well plate (200,000 cells/400 μl per well) with control or β‐blocker treatment. XFe96‐well microplates (Agilent Technologies) were coated with Cell‐Tak (Corning Inc., Corning, NY, USA) beforehand. The day of the assay, cells were washed and incubated in Seahorse RPMI assay medium supplemented with 2 mm glutamine and 1 mm pyruvate (all from Agilent Technologies). Cells were seeded at 60,000 cells per well in pre‐coated XFe96‐well microplates. Oxygen consumption rate (OCR) and extracellular acidification rate (ECAR) were measured in real time using a Mito Stress Test Kit (Agilent Technologies). Oligomycin was injected through ports at 1.5 μm, FCCP at 1.0 μm, and rotenone/antimycin A at 0.5 μm. After the experiment, cells were stained with Hoechst solution (#33258, Sigma‐Aldrich) for normalization. Data were normalized by cell numbers quantified using a Cytation 1 Cell Imaging Reader (Agilent Technologies).

### Glucose measurements

Glucose levels were measured using an Amplex Red Glucose/Glucose Oxidase Assay Kit (Invitrogen, Eugene, OR, USA). LP‐1 and XG‐2 cell lines were seeded in 24‐well plates at 150,000 cells/ml and treated with increasing concentrations of propranolol for 6 and 24 h. For glucose analysis, the sample supernatants were 1/50 diluted in reaction buffer. Glucose concentration was calculated relative to a d‐glucose standard curve and normalized for total cellular protein content as measured using a BCA Protein Assay Kit.

### Statistical analyses

Results and half‐maximal inhibitory concentration (IC_50_) were analyzed using GraphPad Prism 9.1.1 software (GraphPad Software Inc., San Diego, CA, USA). Results are presented as the mean ± SD and were analyzed using the Mantel–Cox test, the Mann–Whitney *U*‐test, and one‐way ANOVA. *p < 0.05, **p < 0.01, ***p < 0.001, and ****p < 0.0001 were considered statistically significant.

## Results

### *ADRB2* expression is associated with poor survival outcome in multiple myeloma

**Figure 1:**
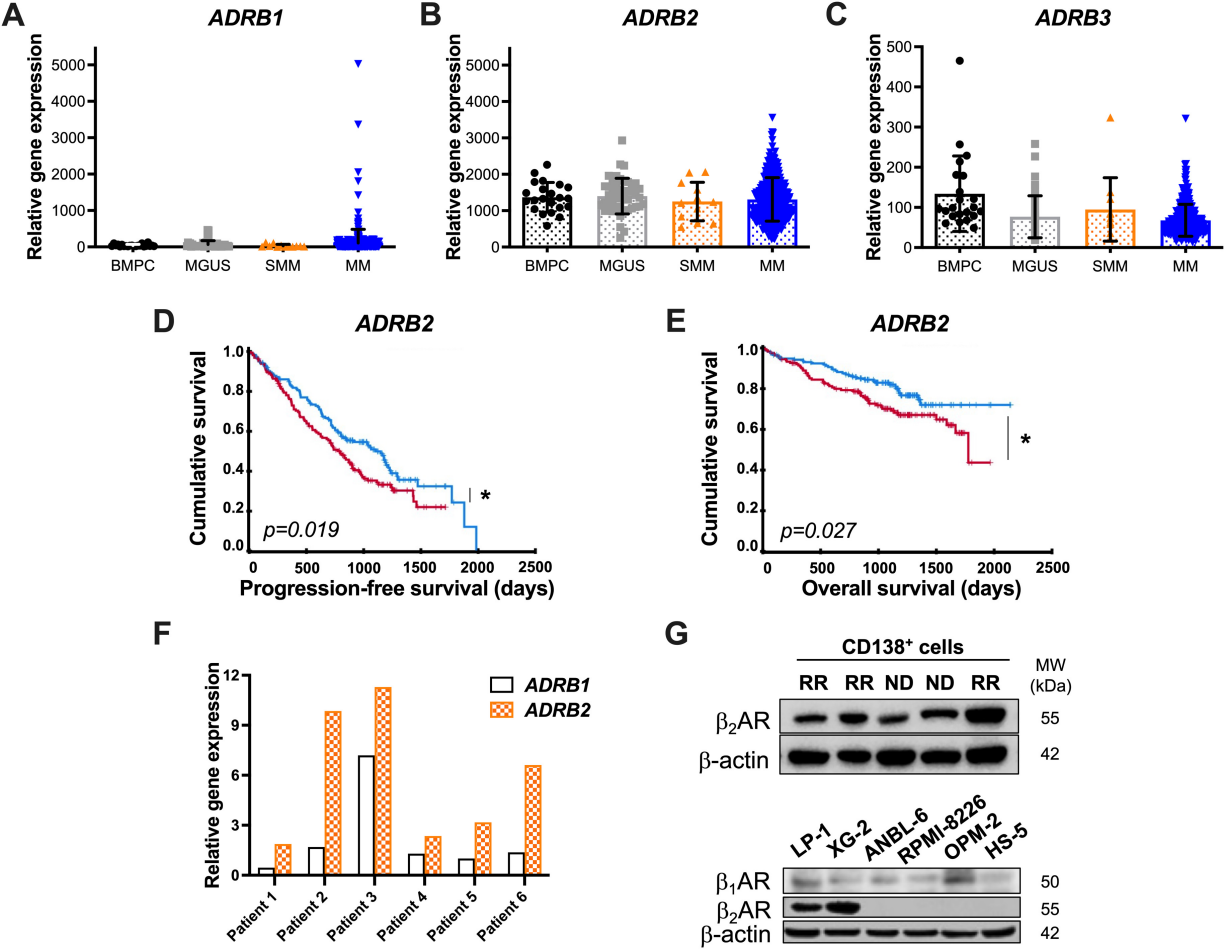
High *ADRB2* expression is correlated with poor survival outcome in multiple myeloma. Gene expression of (A) *ADRB1*, (B) *ADRB2*, and (C) *ADRB3* in healthy BMPCs (*n* = 22), MGUS (*n* = 44), SMM (*n* = 12), and multiple myeloma (MM) cells of newly diagnosed patients (*n* = 345) from the TT2 cohort (accession number GSE2658). (D, E) The prognostic value of *ADRB2* gene expression in newly diagnosed multiple myeloma patients from the MMRF CoMMpass study. A Mantel–Cox test was used to calculate (D) the progression‐free survival and (E) the overall survival curves, with low *ADRB2* expression (blue, *n* = 176) and high *ADRB2* expression (red, *n* = 165). *p < 0.05 is considered statistically significant. (F) RT‐qPCR analysis showing the relative expression of *ADRB1* and *ADRB2* genes in CD138^+^ cells from each patient. (G) Western blot analysis of β_1_AR and β_2_AR protein expression in CD138^+^ cells, derived from five multiple myeloma patients and human multiple myeloma cell lines.

To investigate the expression of β‐adrenergic receptors (*ADRB*) and whether it correlates with patient survival, we consulted several multiple myeloma patient cohorts. The Total Therapy (TT2) cohort data are publicly available and include gene expression profiles of bone marrow plasma cells (BMPCs) of healthy individuals and patients at all stages of MM progression from monoclonal gammopathy of undetermined clinical significance (MGUS) to newly diagnosed multiple myeloma patients. *ADRB1* was expressed at a low level at all stages of disease progression, with a non‐significant increase in multiple myeloma patients (Figure 1A). *ADRB2* was expressed in plasma cells of healthy individuals and at all stages of multiple myeloma progression (MGUS, SMM, MM) (Figure 1B), while *ADRB3* was expressed at low levels (Figure 1C). Of the three βAR subtypes, the *ADRB2* gene was expressed at higher levels in BMPCs and multiple myeloma cells compared with the other subtypes.

To assess the prognostic values of the three *ADRB* subtypes in multiple myeloma patients, we utilized the publicly available MMRF CoMMpass Trial database. The CoMMpass study is a prospective observational study of newly diagnosed multiple myeloma patients who, in contrast to TT2, were exposed to common first‐line treatment regimens including immunomodulatory drugs and proteasome inhibitors. Survival analysis using the Kaplan–Meier method revealed that patients with high *ADRB2* expression had a shorter progression‐free survival (*p* = 0.019) (Figure 1D) and overall survival (*p* = 0.027) (Figure 1E) compared with patients with low *ADRB2* expression (hazard ratio 1.6). In contrast to *ADRB2*, the expression of *ADRB1* and *ADRB3* genes was too low to assess their prognostic value.

To validate the expression levels of β_2_AR in primary multiple myeloma samples, bone marrow‐derived CD138^+^ multiple myeloma cells were purified and analyzed by RT‐qPCR and western blotting. Consistent with the transcriptomics analysis, we observed higher‐level expression of *ADRB2* compared with *ADRB1* (Figure 1F), with β_2_AR protein detectable in all samples (Figure 1G). To investigate the expression levels of *ADRB1*, *ADRB2*, and *ADRB3* in human multiple myeloma cell lines, we consulted the Lombardi and Heidelberg‐Montpellier cohorts (supplementary material, Figure S1). We observed higher *ADRB2* expression compared with *ADRB1* and *ADRB3* in both cohorts. Across human multiple myeloma cell lines, we observed heterogeneous levels of β_2_AR, with the highest levels in LP‐1 and XG‐2, while β_1_AR protein was present at a low level in all cell lines tested (Figure 1G).

### β_2_AR‐blockers decrease the cell viability and key survival pathways in human multiple myeloma cell lines and patient‐derived multiple myeloma cells

**Figure 2:**
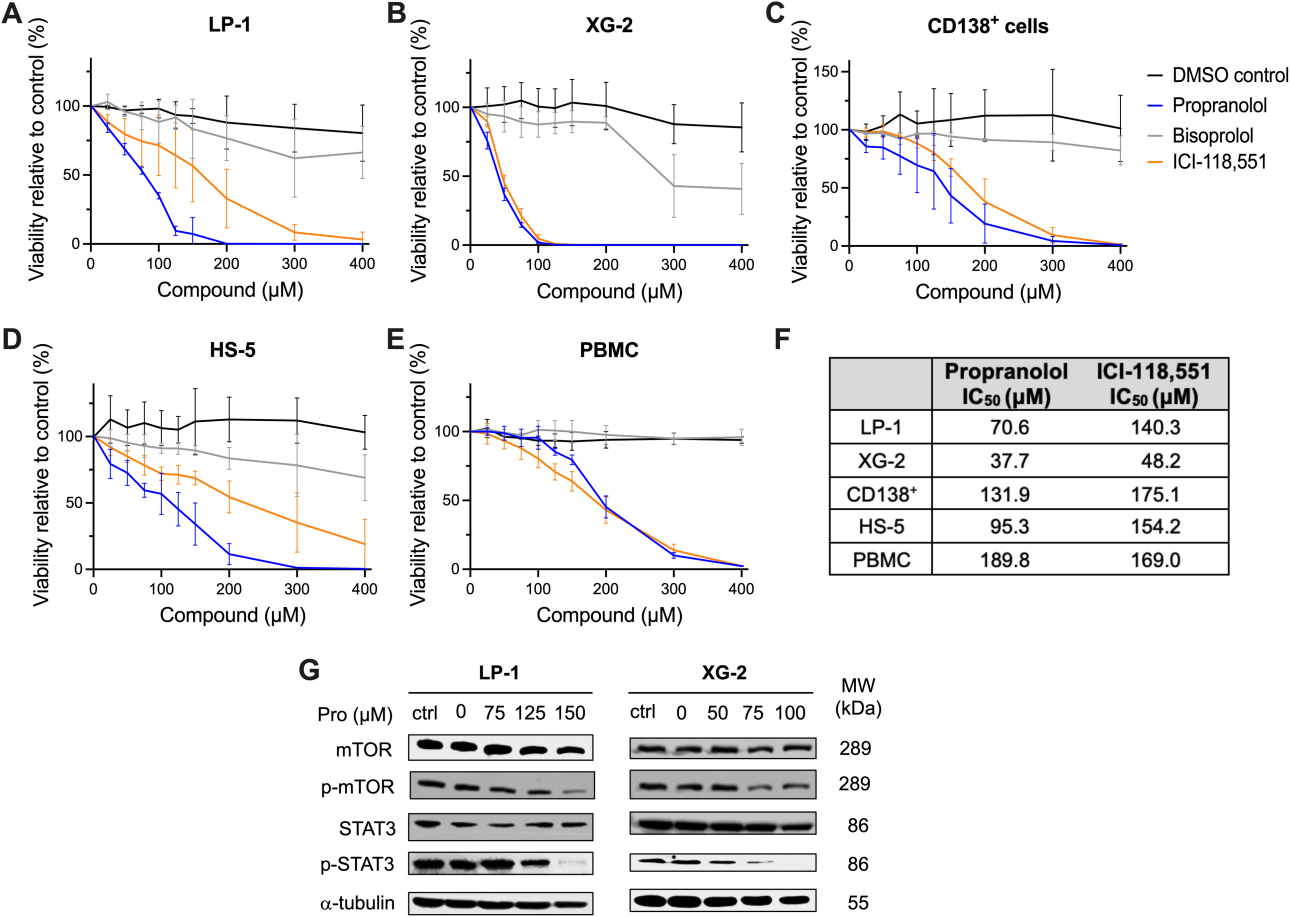
β_2_AR‐blockers decrease the cell viability and key survival pathways in human multiple myeloma cell lines and patient‐derived multiple myeloma cells. Cell lines (A) LP‐1 and (B) XG‐2, and (C) patients’ CD138^+^ cells, (D) stromal cells HS‐5, and (E) PBMCs were treated with propranolol (blue), bisoprolol (gray), ICI‐118,551 (orange), and DMSO (black) at the indicated concentrations for 24 h. Cell viability was analyzed using a CellTiter‐Glo luminescence assay (*n* = 4). (F) For each cell line, the IC_50_ values (μm) were determined based on the dose–response curves (A–E). (G) Western blot analysis of markers involved in survival pathways such as mTOR and STAT3 in LP‐1 and XG‐2 cells following 24 h exposure to increasing concentrations of propranolol. The untreated condition (Ctrl), as well as 0 μm propranolol which includes DMSO treatment for LP‐1 and XG‐2, was used as a control. All markers were analyzed for five independent experiments; one experiment is shown. α‐Tubulin was used as a loading control.

Cardio‐selective β_1_AR‐blockers are dominantly used in clinical practice. However, we observed that the β_2_AR is a poor prognostic factor in multiple myeloma (Figure 1D,E). Therefore, we assessed the anti‐tumor effects of targeting either β_1_, β_2_, or both βARs as a therapeutic option in multiple myeloma. To investigate anti‐myeloma effects, we chose β_2_AR‐expressing LP‐1 and XG‐2 cell lines as representative of the human situation (Figure 1A–C,G). LP‐1 and XG‐2 cell lines were treated with the selective β_1_AR‐blocker bisoprolol, the selective β_2_AR‐blocker ICI‐118,551, and the non‐selective β‐blocker propranolol for 24 h. Cells were treated with different concentrations (ranging from 0 to 400 μm) of β‐blockers, or DMSO as a vehicle control. β_2_AR targeting by non‐selective propranolol and selective ICI‐118,551 caused a dose‐dependent decrease in the cell viability of multiple myeloma cells (Figure 2A,B). In contrast, β_1_AR targeting using bisoprolol had no effect on the viability of multiple myeloma cells, even at the highest concentrations.

We then evaluated the effect of β‐blockers on primary multiple myeloma samples by treating bone marrow‐derived CD138^+^ multiple myeloma cells. Consistent with the results obtained in human multiple myeloma cell lines, treatment with β_2_AR‐blockers reduced the viability of primary multiple myeloma cells (Figure 2C). To investigate if β‐blockers had a similar effect on the viability of healthy cells, we treated the HS‐5 cell line, a fibroblast‐like cell line derived from healthy bone marrow, with β‐blockers. β_2_AR‐blockers reduced the viability of HS‐5, but to a lesser extent than the effects observed in multiple myeloma cells (Figure 2D). To further elucidate the effect of β‐blockers on healthy cells, we treated peripheral blood mononuclear cells (PBMCs) with β‐blockers. β_2_AR‐blockers reduced the viability of PBMCs at higher concentrations (Figure 2E). For each cell line and the CD138^+^ multiple myeloma cells and PBMCs, we calculated the IC_50_ values based on the dose–response curves (Figure 2F). The IC_50_ values of propranolol for HS‐5 cells and PBMCs were higher (95.3 μm for HS‐5 cells and 189.8 μm for PBMCs) compared with the multiple myeloma cell lines (37.7–70.6 μm). Similar results were obtained with the selective β_2_AR‐blocker (48.2–140.3 μm for myeloma cells versus 154.2 μm for HS‐5 cells and 169.0 μm for PBMCs).

To further understand the anti‐survival effects of β_2_AR‐blockers, we studied signaling pathways that are known be involved in multiple myeloma cell survival. Western blot analysis of the mTOR and STAT3 survival pathways showed a significant reduction in p‐mTOR and p‐STAT3 in both LP‐1 and XG‐2 multiple myeloma cells treated with propranolol (Figure 2G and supplementary material, Figure S2). Taken together, our data show that β_2_AR targeting affects multiple myeloma cell viability and decreases the associated survival pathways.

### Propranolol induces apoptosis and autophagy in β_2_AR‐expressing human multiple myeloma cell lines

**Figure 3:**
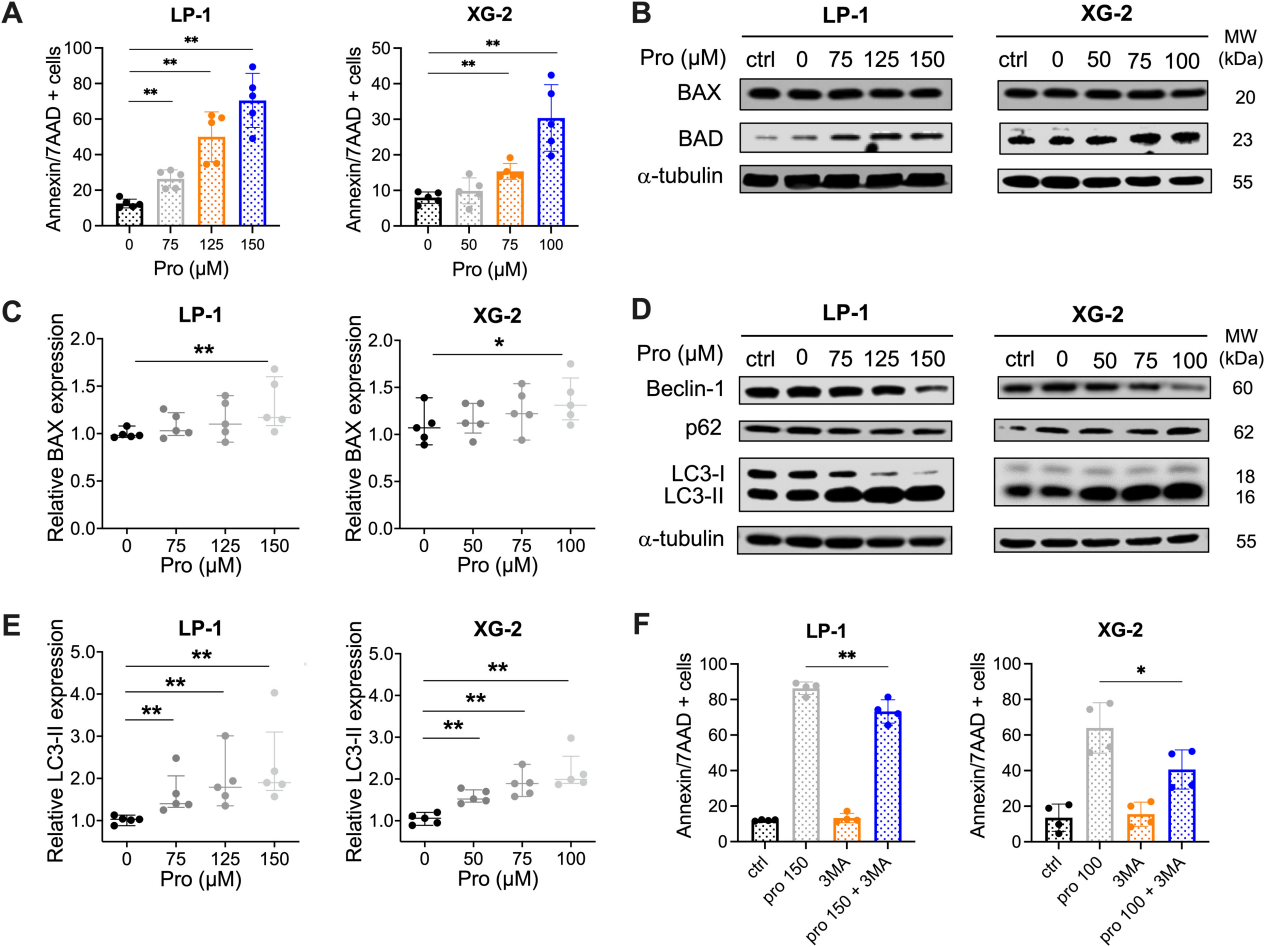
Propranolol induces apoptosis and autophagy in β_2_AR‐expressing human multiple myeloma cell lines. (A) LP‐1 and XG‐2 cells were subjected to concentrations of propranolol for 24 h. Apoptosis was determined by flow cytometry using an annexin V/7‐AAD staining method. Mann–Whitney *U*‐test, with *p < 0.05 considered statistically significant (*n* = 5). (B) Western blot analysis of the pro‐apoptotic marker BAX and BAD following 24 h propranolol treatment of LP‐1 and XG‐2 cells. (C) Quantification of BAX using ImageJ software. (D) Autophagic activity following propranolol treatment was analyzed by western blotting in LP‐1 and XG‐2 cells, using autophagic markers Beclin‐1, p62, and LC3‐I/‐II. α‐Tubulin was used as a loading control. The untreated condition (Ctrl), as well as 0 μm propranolol which includes DMSO treatment for LP‐1 and XG‐2, was used as a control. All markers were analyzed for five independent experiments; one experiment is shown. (E) Quantification of LC3‐I/‐II using ImageJ software. Statistical analysis was performed using a one‐sided Mann–Whitney *U*‐test, with *p < 0.05 and **p < 0.01 considered statistically significant (*n* = 5). LP‐1 and XG‐2 cells, pretreated with 2 mm 3MA for 4 h, 150 and 100 μm propranolol, respectively, were added to the culture medium. (F) Apoptosis was determined by flow cytometry using an annexin V/7‐AAD staining method. Statistical analysis was performed using one‐way ANOVA; **p < 0.01 and *p < 0.05 were considered statistically significant compared with control (*n* = 4).

As our findings demonstrated a more potent reduction in the cell viability of multiple myeloma cells using propranolol compared with the selective β_2_AR‐blocker ICI‐118,551 (Figure 2A–F), we further elucidated the anti‐myeloma effects of the non‐selective β_2_AR‐blocker propranolol. Propranolol is a clinically used drug for the treatment of cardiovascular disease, with a well‐known safety profile. To investigate whether the effects on cell viability (Figure 2) were associated with induction of apoptosis, LP‐1 and XG‐2 cells were treated with different concentrations of propranolol and apoptosis was investigated after 24 h by flow cytometry using the annexin V/7‐AAD staining method. Propranolol significantly induced apoptosis in LP‐1 and XG‐2 cells (Figure 3A) and increased the pro‐apoptotic marker BAX (Figure 3B,C). In addition, propranolol induced both intrinsic and extrinsic apoptosis, as illustrated by increased levels of cleaved caspase‐3, caspase‐8 (extrinsic), and caspase‐9 (intrinsic) (supplementary material, Figure S3A).

Accumulating evidence supports a potential role for β_2_AR signaling in autophagy regulation [18, 19]. Besides its protective role, upregulation of autophagy promotes cell death. Hence, we aimed to investigate the effect of the non‐selective β‐blocker propranolol on autophagy by analyzing three central autophagy‐related proteins: LC3‐II, p62, and Beclin‐1 [20]. Propranolol stimulated the conversion of the soluble LC3‐I to non‐soluble LC3‐II, which is considered as a quantitative indicator of autophagosome formation (Figure 3D). LC3‐II expression was significantly upregulated, indicating excessive autophagosome formation or autophagy‐related structures (Figure 3E). Additionally, we observed an increase in p62 levels (supplementary material, Figure S3B), while Beclin‐1 remained stable. p62 is incorporated into the completed autophagosomes but degraded in autolysosomes [21]. Therefore, our findings suggest that propranolol treatment induces an imbalance between autophagosome formation and lysosomal degradation, resulting in cellular dysfunction and cell death. To further investigate the involvement of autophagy in propranolol‐mediated apoptosis, we pretreated LP‐1 and XG‐2 cells for 4 h with 2 mm autophagy inhibitor 3‐methyladenine (3MA). Propranolol was then added to the cells and apoptosis was investigated after 24 h by flow cytometry using the annexin V/7‐AAD staining method. Apoptosis was significantly decreased in the LP‐1 and XG‐2 cells treated with the combination of 3MA and propranolol (Figure 3F). Moreover, the combination of propranolol with 3MA significantly decreased the LC3‐II/I ratio and p62 levels, indicating that 3MA could block the propranolol‐induced autophagy (supplementary material, Figure S3C–E). These findings imply that propranolol‐mediated apoptosis of multiple myeloma cells is partially mediated by the induction of autophagy.

### β_2_AR‐blockers decrease mitochondrial respiration and glycolytic activity in multiple myeloma cells

**Figure 4:**
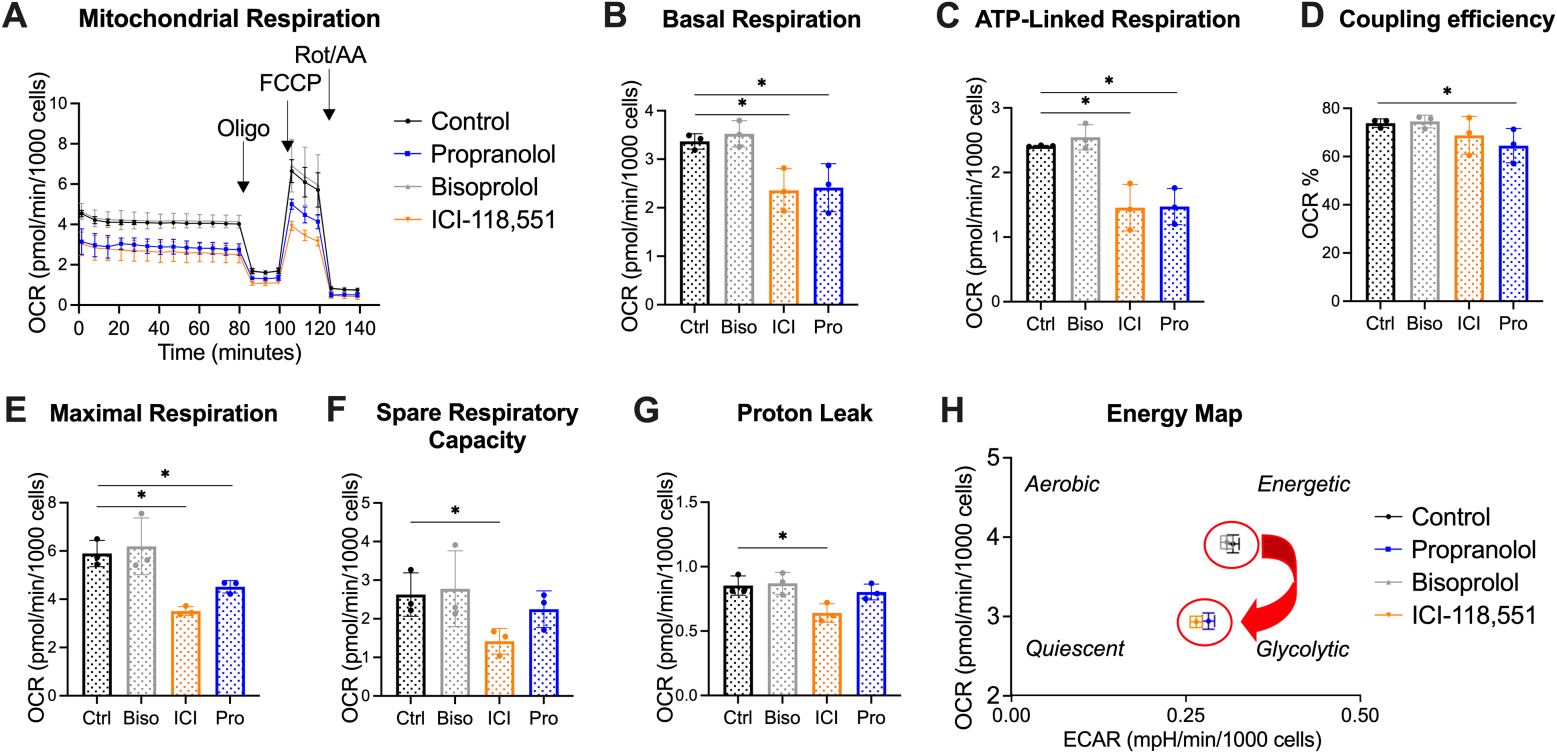
β_2_AR‐blockers decrease mitochondrial respiration. LP‐1 cells were treated with 50 μm bisoprolol (Biso, gray), ICI‐118,551 (ICI, orange), or propranolol (Pro, blue) for 24 h. DMSO (black)‐treated cells were used as a control (Ctrl). Mitochondrial bioenergetics was analyzed using the Agilent XF Seahorse technology. (A) Mitochondrial respiration, (B) basal respiration, (C) ATP‐linked respiration, (D) coupling efficiency, (E) maximal respiration, (F) spare respiratory capacity, (G) proton leak, and (H) energy map were calculated following three injections of the mitochondria test kit: oligo (1.5 μm), FCCP (1 μm), and Rot/AA (0.5 μm). Mean ± SD. Statistical analysis was performed using a Mann–Whitney *U*‐test; *p < 0.05 compared with control (*n* = 3). Oligo: oligomycin; FCCP: carbonyl cyanide 4‐(trifluoromethoxy)phenylhydrazone; Rot/AA: rotenone/antimycin A.

Besides induction of apoptosis and autophagy, a number of studies suggest that β‐blockers might alter tumor cell metabolism (e.g. glycolysis, lipid metabolism) [22, 23, 24]. As mitochondrial activity and glycolysis are associated with disease progression in multiple myeloma [25], we assessed the impact on multiple myeloma cell metabolism of βAR‐blockers that target β_1_, β_2_, or both subtypes. Using the Seahorse analyzer, we measured the oxygen consumption rate (OCR) as an indicator of mitochondrial respiration and the extracellular acidification rate (ECAR), which is largely the result of glycolytic activity. LP‐1 cells express both β_1_AR and β_2_AR. The non‐selective β‐blocker propranolol and β_2_AR‐blocker ICI‐118,551 decreased mitochondrial respiration in LP‐1 cells, while no effect was observed using the β_1_AR‐blocker bisoprolol (Figure 4A). Treatment with propranolol or ICI‐118,551 significantly decreased basal respiration, ATP‐linked respiration, and maximal respiration (Figure 4B,C,E). Moreover, propranolol treatment significantly reduced the coupling efficiency (Figure 4D), while ICI‐118,551 decreased the spare respiratory capacity and the mitochondrial proton leak (Figure 4F,G). As shown in Figure 4H, we found that the impact of β_2_‐blockade on OCR and ECAR shifted the energetic phenotype of multiple myeloma cells to less glycolytic and more quiescent. Similar effects were observed after 48 h β‐blocker treatment (supplementary material, Figure S4).

**Figure 5:**
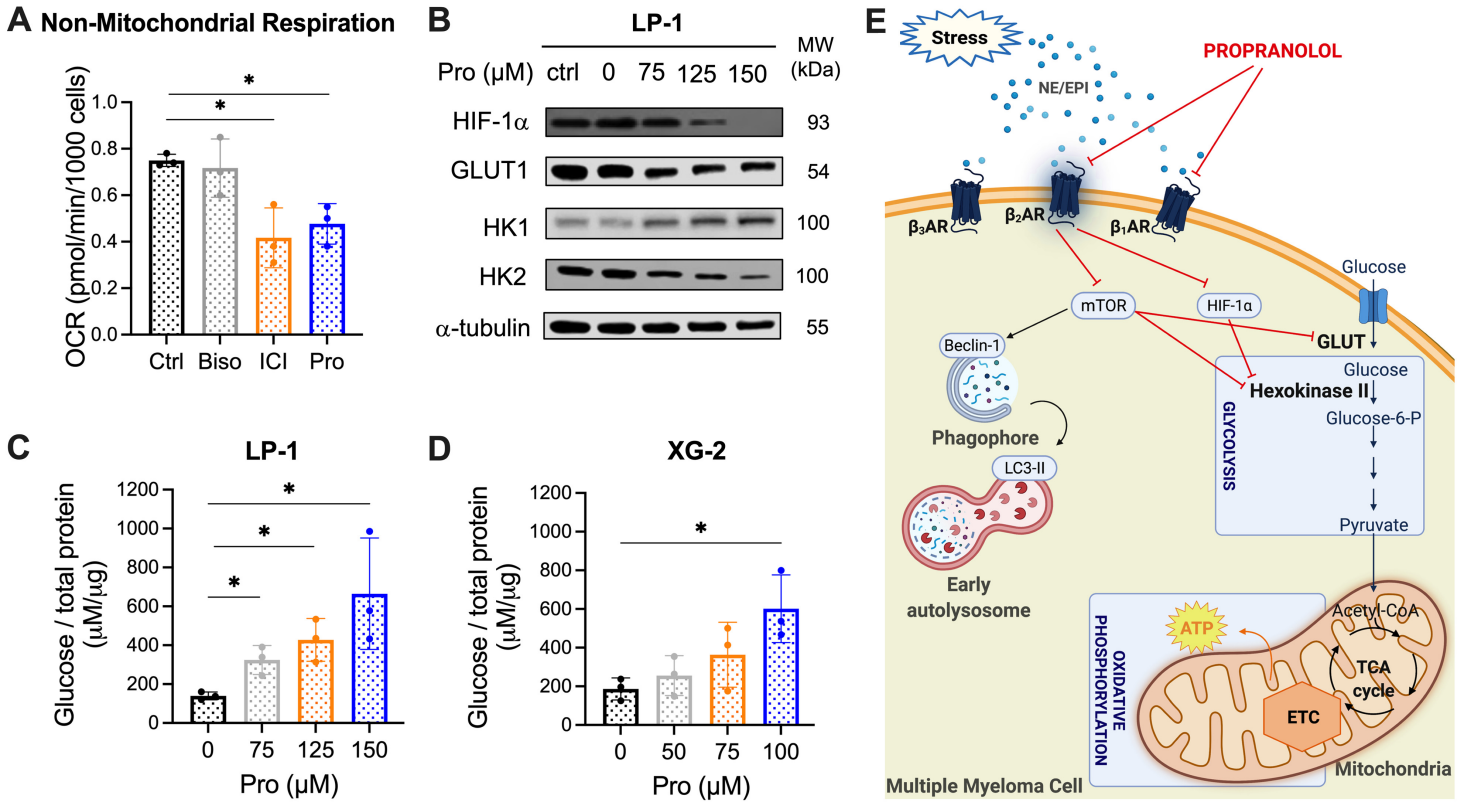
β_2_AR‐blockers decrease glycolytic activity in multiple myeloma cells. (A) Non‐mitochondrial respiration after 24 h β‐blocker treatment, measured using a Seahorse analyzer. (B) Western blot analysis of metabolic markers involved in the glycolytic pathway HIF‐1α, GLUT1, HK1, and HK2 expression in LP‐1 cells following 24 h exposure to propranolol. All markers were analyzed for five independent experiments; one experiment is shown. α‐Tubulin was used as a loading control. (C, D) Glucose levels measured using the Amplex Red glucose/glucose oxidase assay kit. Supernatant of LP‐1 and XG‐2 cells was collected after 24 h propranolol treatment. Mann–Whitney *U*‐test, with *p < 0.05 considered statistically significant (*n* = 3). (E) Graphical abstract of β_2_AR‐blocker‐mediated effects, created with Biorender.com. NE: norepinephrine; EPI: epinephrine; TCA: tricarboxylic acid cycle; ETC: electron transport chain.

Glycolysis is the primary process by which cells break down glucose and generate ATP. As enhanced glycolysis in multiple myeloma cells has been linked to tumor cell survival and drug resistance, we further elucidated the impact of β‐blocker therapy on this process [26]. Seahorse analysis revealed that blockade of β_2_AR, but not β_1_AR, resulted in a significant reduction in non‐mitochondrial respiration, which includes glycolysis (Figure 5A). To further validate the impact of β_2_AR‐blockers on glycolysis, a process that is particularly upregulated under hypoxic conditions, we investigated downstream glycolytic enzymes. Propranolol decreased hypoxia‐inducible factor 1‐alpha (HIF‐1α) in LP‐1 and XG‐2 cells, and the HIF‐1α responsive glucose transporter 1 (GLUT1) in XG‐2 cells. In addition, β‐blockade significantly decreased hexokinase‐II (HK2) in both LP‐1 and XG‐2 cells (Figure 5B and supplementary material, Figure S5B–F), but increased hexokinase‐I (HK1). This increase in HK1 might suggest a potential compensatory effect on propranolol‐mediated metabolic inhibition in multiple myeloma cells. The hexokinase family is responsible for the initial step in glycolysis, by catalyzing the phosphorylation of glucose to glucose‐6‐phospate. The isoform HK1 is found in a variety of tissues, while HK2 is present in pathological conditions. Pyruvate kinase M2 (PKM2) and lactate dehydrogenase A (LDHA), which are also involved in the glycolytic pathway, were not affected by propranolol treatment (data not shown). We further investigated the effects of propranolol on glucose transport. Propranolol decreased cellular glucose uptake at 24 h, as demonstrated by significantly increased free glucose in the supernatant of LP‐1 and XG‐2 cells (Figure 5C,D and supplementary material, Figure S5I).

To better understand the impact of *GLUT1* and *HK2* downregulation in multiple myeloma pathogenesis, we determined the expression of both genes in multiple myeloma patients using the TT2 cohort. Both genes were expressed in plasma cells of healthy individuals and at all stages of disease progression (MGUS, SMM, MM) (supplementary material, Figure S5G,H). Using the MMRF CoMMpass study, we found that high *GLUT1* and *HK2* expression was correlated with poor overall survival in multiple myeloma patients (*GLUT1*: *p* = 0.022; *HK2*: *p* = 0.034) (supplementary material, Figure S5J,K).

Taken together, these data demonstrate that β_2_AR‐blockers affect glycolytic activity by reducing GLUT1 and HK2. These two key regulators of cell metabolism are downstream effectors of the multiple myeloma survival pathway mTOR. In addition to glycolysis, β_2_AR‐blockers also decreased the mitochondrial respiration of multiple myeloma cells (Figure 5E).

### Propranolol sensitizes multiple myeloma cells to bortezomib and melphalan

**Figure 6:**
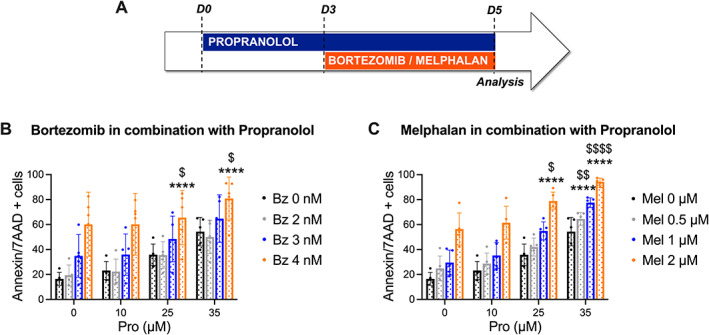
Propranolol in combination with standard‐of‐care agents increased apoptosis. (A) Schematic overview of the experimental design. Apoptosis was analyzed by flow cytometry using an annexin V/7‐AAD staining method. LP‐1 cells were treated with 10, 25, or 35 μm propranolol (Pro) for 5 days; at day 3, bortezomib (Bz, 2, 3, or 4 nm) or melphalan (Mel, 0.5, 1, or 2 μm) was added to the cells. DMSO‐treated cells were used as a control (Ctrl). (B, C) Quantitative analysis of apoptotic cells. Mean ± SD. Statistical analysis was performed using one‐way ANOVA; ****p < 0.0001 and *p < 0.05 were considered statistically significant compared with single propranolol treatment and ^$$$$^ *p* < 0.0001, ^$$^ *p* < 0.01, and ^$^ *p* < 0.05 were considered statistically significant compared with single bortezomib or melphalan treatment (*n* = 5).

Increased glycolytic activity has been identified as a potential contributor to multiple myeloma cell drug resistance. Increased glycolysis was previously associated with resistance to the chemotherapeutic agent melphalan [27] and the proteasome inhibitor bortezomib [28, 29], both standard‐of‐care agents in the treatment of multiple myeloma patients. To assess whether β_2_AR‐blockers could potentiate the anti‐tumor effect of either melphalan or bortezomib *in vitro*, LP‐1 cells were pretreated for 3 days at a low concentration of propranolol (10, 25, or 35 μm) and then bortezomib or melphalan was added to the culture for 48 h (Figure 6A). The highest concentration of propranolol induced up to 50% apoptosis as a single agent. In combination with standard therapies, we observed a significant increase in apoptotic cells (Figure 6B,C and supplementary material, Figure S6A–F). We calculated the combination index using the Chou–Talalay method to determine the drug interaction. Propranolol in combination with all the tested melphalan concentrations (0.5, 1, 2 μm) resulted in synergism (supplementary material, Figure S6G). In conclusion, our data illustrate that propranolol may sensitize multiple myeloma cells to standard‐of‐care agents.

## Discussion

Our study identifies the β_2_AR subtype as a poor prognostic factor in multiple myeloma patients. We demonstrated that β‐blockers that target the β_2_AR affect multiple myeloma cell viability and induce apoptosis and autophagy. Additionally, we observed a significant impact of β_2_AR antagonism on multiple myeloma cell metabolism, as demonstrated by reduced mitochondrial respiration and glycolytic activity (Figure 5C). Besides single‐agent activity, we provided evidence that targeting β_2_AR can potentiate the anti‐tumor effects of bortezomib and melphalan in multiple myeloma cells, identifying the potential of propranolol to enhance the effect of current standard‐of‐care agents in multiple myeloma.

Our findings expand the current understanding of how β‐blockers impact cancer cell processes, particularly in the context of multiple myeloma. Previous work in solid and hematological cancers was based on retrospective epidemiological studies that demonstrated a positive impact of β‐blocker therapy on cancer patient survival [8, 9, 10, 11, 12, 13]. However, it remained unclear which βAR was responsible for this phenomenon. In our study, we provide a direct comparison between the β_1_AR‐selective bisoprolol, the β_2_AR‐selective ICI‐118,551, and the non‐selective β‐blocker propranolol. Our data clearly demonstrated that either selective or non‐selective targeting of the β_2_AR subtype is important to exert anti‐tumor effects in multiple myeloma. We observed a heterogeneous βAR expression and response to β‐blocker therapy in the tested multiple myeloma patients’ samples, which could potentially be attributed to differences in disease stage, tumor progression, and therapy, as already reported for other cancer types [30, 31].

Previous studies already indicate an effect of β‐blockers on apoptosis [5, 6], autophagy [18, 19], or cancer cell metabolism [32, 33]; however, our data on mitochondrial respiration and glycolysis provide new mechanistic insights and indicate a potential link between β‐adrenergic signaling, hypoxia, and tumor cell metabolism in multiple myeloma cells. While the current study focused exclusively on the effects of β‐blockers on multiple myeloma cells, a number of studies have demonstrated effects of β‐blocker therapy on angiogenesis [34] and the immune microenvironment including myeloid‐derived suppressor cells [35] and regulatory T cells [36]. As the immune compartment is a key component of the suppressive bone microenvironment in multiple myeloma, additional studies in immunocompetent multiple myeloma models are required to assess the effect of β‐blockers on the different cell populations and fully understand their mechanism of action.

Our finding that propranolol potentiates the anti‐tumor effect of first‐line therapies bortezomib and melphalan supports the evaluation of this β‐blocker in a therapeutic setting in multiple myeloma patients. As glycolytic activity has been linked with bortezomib [28, 29] and melphalan resistance [27], β_2_AR‐blockers may be useful in patients who have acquired resistance to these drugs. Studies in solid tumor models provided some evidence of using propranolol as a chemo‐ or radio‐sensitizer [22, 37], or to improve the outcome of cancer immunotherapies (e.g. anti‐CTLA4 therapy) [38]. Propranolol is a widely used β‐blocker with a well‐known toxicity profile, which may allow rapid and affordable clinical implementation. The study of Knight *et al* demonstrated the feasibility of propranolol administration in multiple myeloma patients who received an autologous hematopoietic stem cell transplantation [16]. Current clinical trials (e.g. NCT04848519, NCT04493489) that test propranolol as an adjuvant therapy in cancer will help to determine the optimal dose and treatment schedule to initiate a phase I clinical trial in multiple myeloma patients.

While selective β_1_‐blockers are commonly prescribed to treat cardiovascular disease, the findings of this study underscore the potential of drugs that target the β_2_AR as an adjunct therapy in multiple myeloma patients. Our data support the evaluation of propranolol as an adjuvant chemosensitizer in multiple myeloma in a randomized clinical trial.

## Author contributions statement

HS, EKS and KDV contributed to the design of the study. HS, EV, PV, GA and KDV collected and interpreted data. Material was provided by NDB and JM. The study was supervised by KDV. The manuscript was written by HS and KDV and was revised and approved by all the authors.

## Supporting information


**Figure S1.** Transcriptome analysis of β‐adrenergic receptors in the Lombardi and Heidelberg‐Montpellier cohorts
**Figure S2.** Quantification of survival pathways after propranolol treatment
**Figure S3.** Propranolol induces intrinsic and extrinsic apoptosis and autophagy in β_2_AR‐expressing human multiple myeloma cell lines
**Figure S4.** β_2_AR‐blockers decrease mitochondrial respiration
**Figure S5.** Quantification of metabolic pathways after propranolol treatment and prognostic value of metabolic markers in multiple myeloma
**Figure S6.** Propranolol in combination with standard‐of‐care agents increased apoptosis and resulted in synergistic effects

## Data Availability

Data analyzed in this study were a re‐analysis of existing data, which are openly available at locations cited in the References section.
