# Supplementary material for: Targeting the β2‐adrenergic receptor increases chemosensitivity in multiple myeloma by induction of apoptosis and modulating cancer cell metabolism
Source: J Pathol. 2022 Nov 22;259(1):69–80. doi: 10.1002/path.6020 (PMC10953387; doi:10.1002/path.6020)
Supplement: Supplementary file 1 — Figure S1. Transcriptome analysis of β‐adrenergic receptors in the Lombardi and Heidelberg‐Montpellier cohorts Figure S2. Quantification of survival pathways after propranolol treatment Figure S3. Propranolol induces intrinsic and extrinsic apoptosis and autophagy in β2AR‐expressing human multiple myeloma cell lines Figure S4. β2AR‐blockers decrease mitochondrial respiration Figure S5. Quantification of metabolic pathways after propranolol treatment and prognostic value of metabolic markers in multiple myeloma Figure S6. Propranolol in combination with standard‐of‐care agents increased apoptosis and resulted in synergistic effects [file PATH-259-69-s001.docx]

**Targeting the β_2_-adrenergic receptor increases chemosensitivity in multiple myeloma by induction of apoptosis and modulating cancer cell metabolism**

H Satilmis *et al. J Pathol* <https://doi.org/10.1002/path.6020>

**Supplementary Figures S1–S6**

**
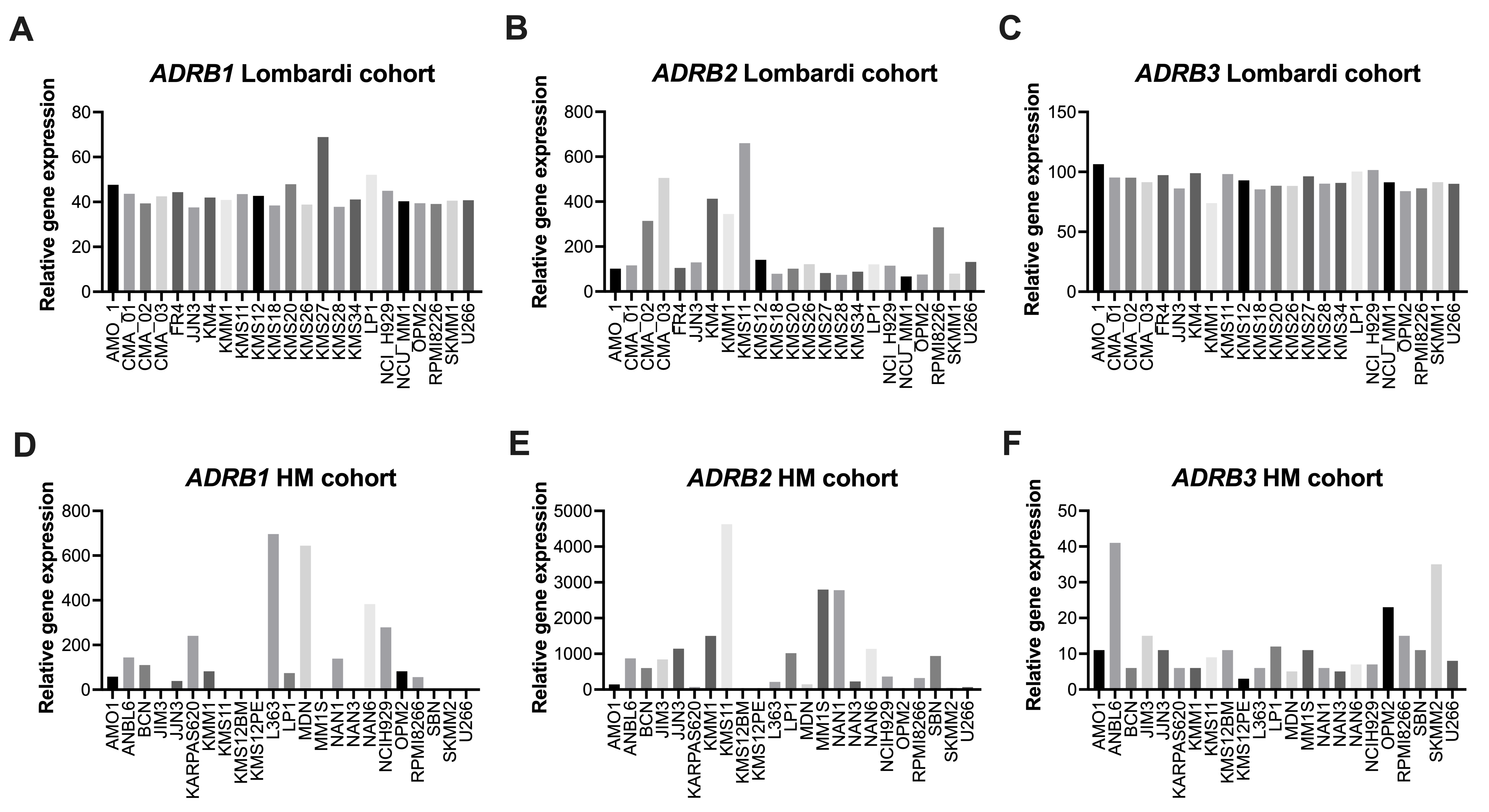
**

**Figure S1. Transcriptome analysis of β-adrenergic receptors in the Lombardi and Heidelberg-Montpellier cohorts.**

(A–F) Gene expression of *ADRB1*, *ADRB2*, and *ADRB3* in multiple myeloma cell lines from the Lombardi cohort (*n* = 23) (A–C) and the Heidelberg-Montpellier cohort (*n* = 23) (D–F). Gene expression data were normalized using the MAS5 algorithm.


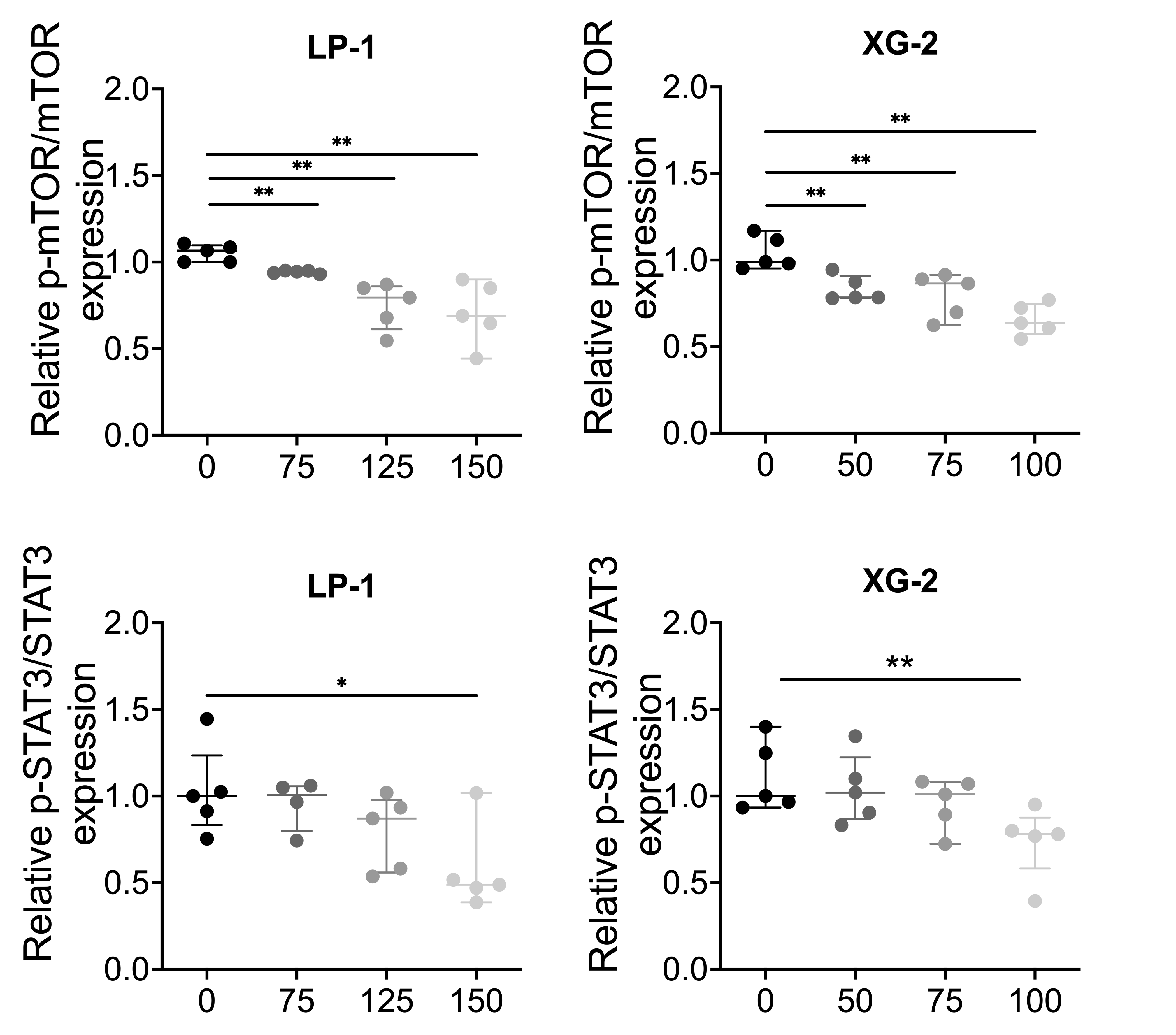


**Figure S2. Quantification of survival pathways after propranolol treatment.**

Quantification of western blot analysis of p-mTOR/mTOR and p-STAT3/STAT3 using ImageJ software. Statistical analysis was performed using a one-sided Mann–Whitney *U*-test, with *p* < 0.05 (*) and *p* < 0.01 (**) considered statistically significant (*n* = 5).

**
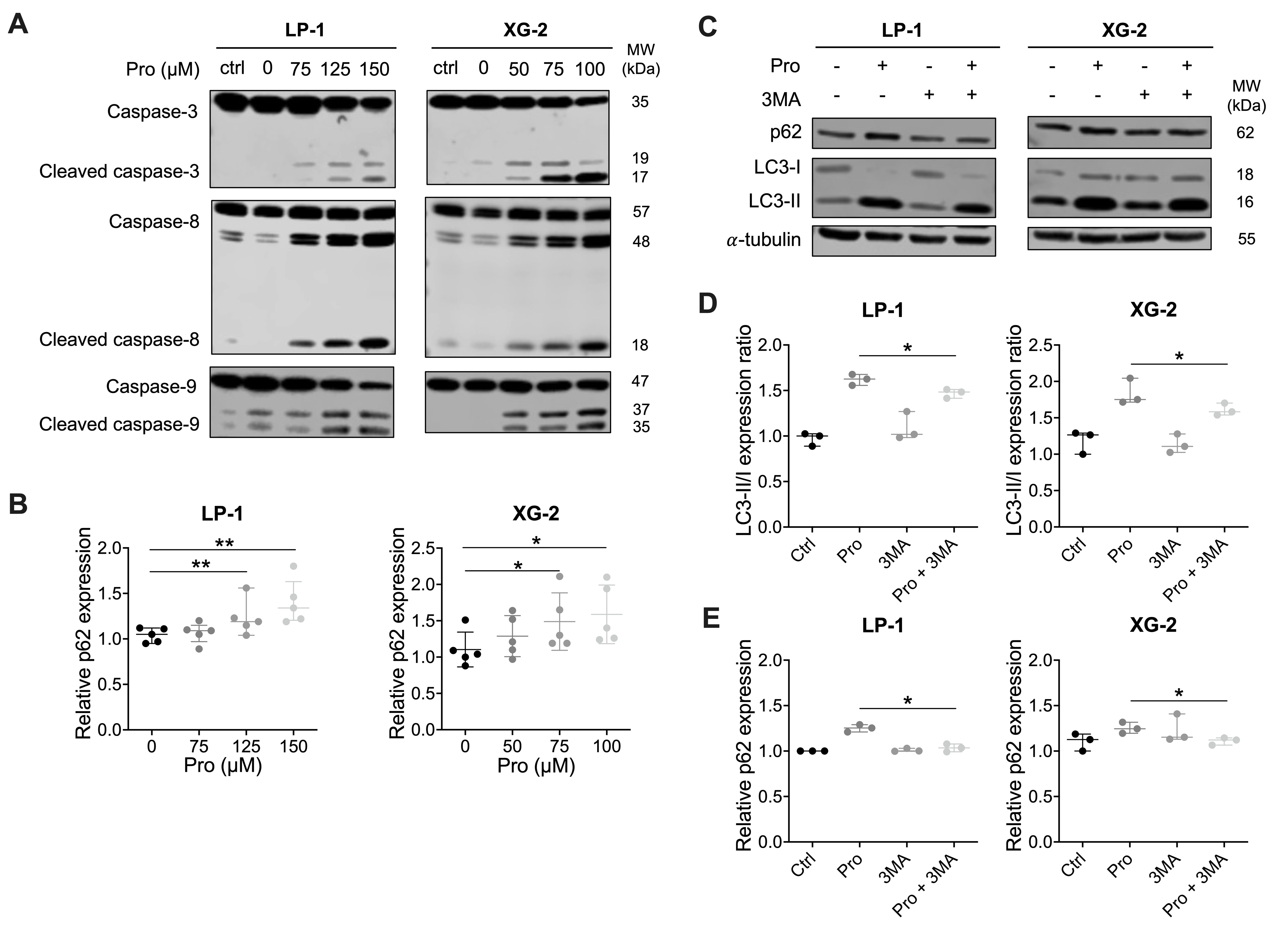
**

**Figure S3. Propranolol induces intrinsic and extrinsic apoptosis and autophagy in** β**_2_AR-expressing human multiple myeloma cell lines.**

(A) Western blot analysis of caspases following 24 h propranolol treatment of LP-1 and XG-2 cells. All markers were analyzed for five independent experiments; one experiment is shown. (B) Quantification of western blot analysis of p62 using ImageJ software. Statistical analysis was performed using a one-sided Mann–Whitney *U*-test, with *p* < 0.05 (*) and *p* < 0.01 (**) considered statistically significant (*n* = 5). (C) Western blot analysis of p62, LC3-I/-II of LP-1 and XG-2 cells treated with the combination of 2 mm 3MA and propranolol. All markers were analyzed for three independent experiments; one experiment is shown. α-Tubulin was used as a loading control. (D, E) Quantification of western blot analysis of LC3-II/-I ratio (D) and p62 (E) using ImageJ software. Statistical analysis was performed using a one-sided Mann–Whitney *U*-test, with *p* < 0.05 (*) considered statistically significant (*n* = 5).


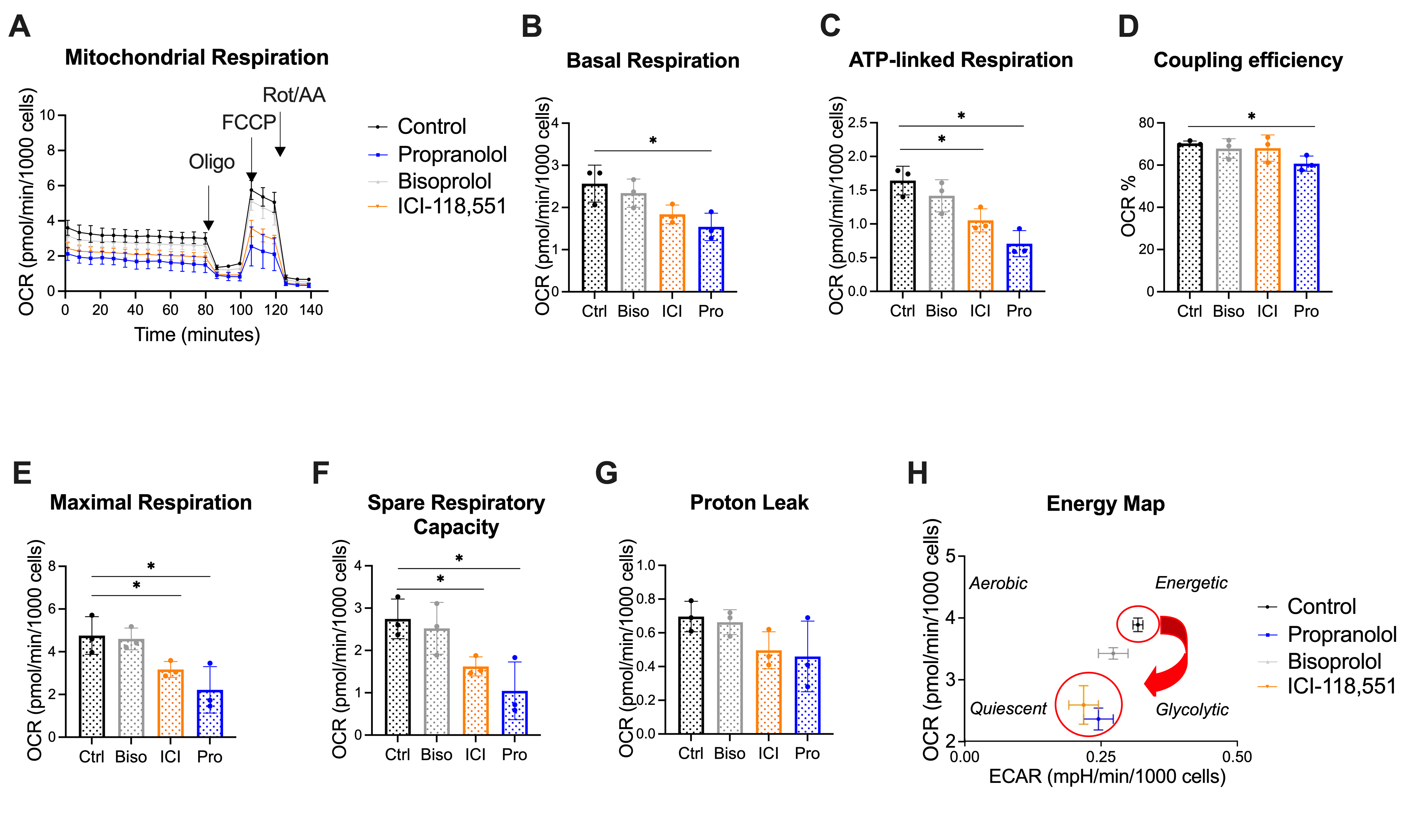


**Figure S4. β_2_AR-blockers decrease mitochondrial respiration.**

LP-1 cells were treated with 50 μm bisoprolol (Biso, gray), ICI-118,551 (ICI, orange), or propranolol (Pro, blue) for 48 h. DMSO (black)-treated cells were used as a control (Ctrl). Mitochondrial bioenergetics was analyzed using the Agilent XF Seahorse technology. (A) Mitochondrial respiration, (B) basal respiration, (C) ATP-linked respiration, (D) coupling efficiency, (E) maximal respiration, (F) spare respiratory capacity, (G) proton leak, and (H) energy map (H) were calculated following three injections of the mitochondrial test kit: Oligo (1.5 μm), FCCP (1 μm), and Rot/AA (0.5 μm). Mean ± SD. Statistical analysis was performed using a Mann–Whitney *U*-test, with * indicating *p* < 0.05 compared with control (*n* = 3). Oligo: oligomycin; FCCP: carbonyl cyanide 4-(trifluoromethoxy)phenylhydrazone; Rot/AA: rotenone/antimycin A.


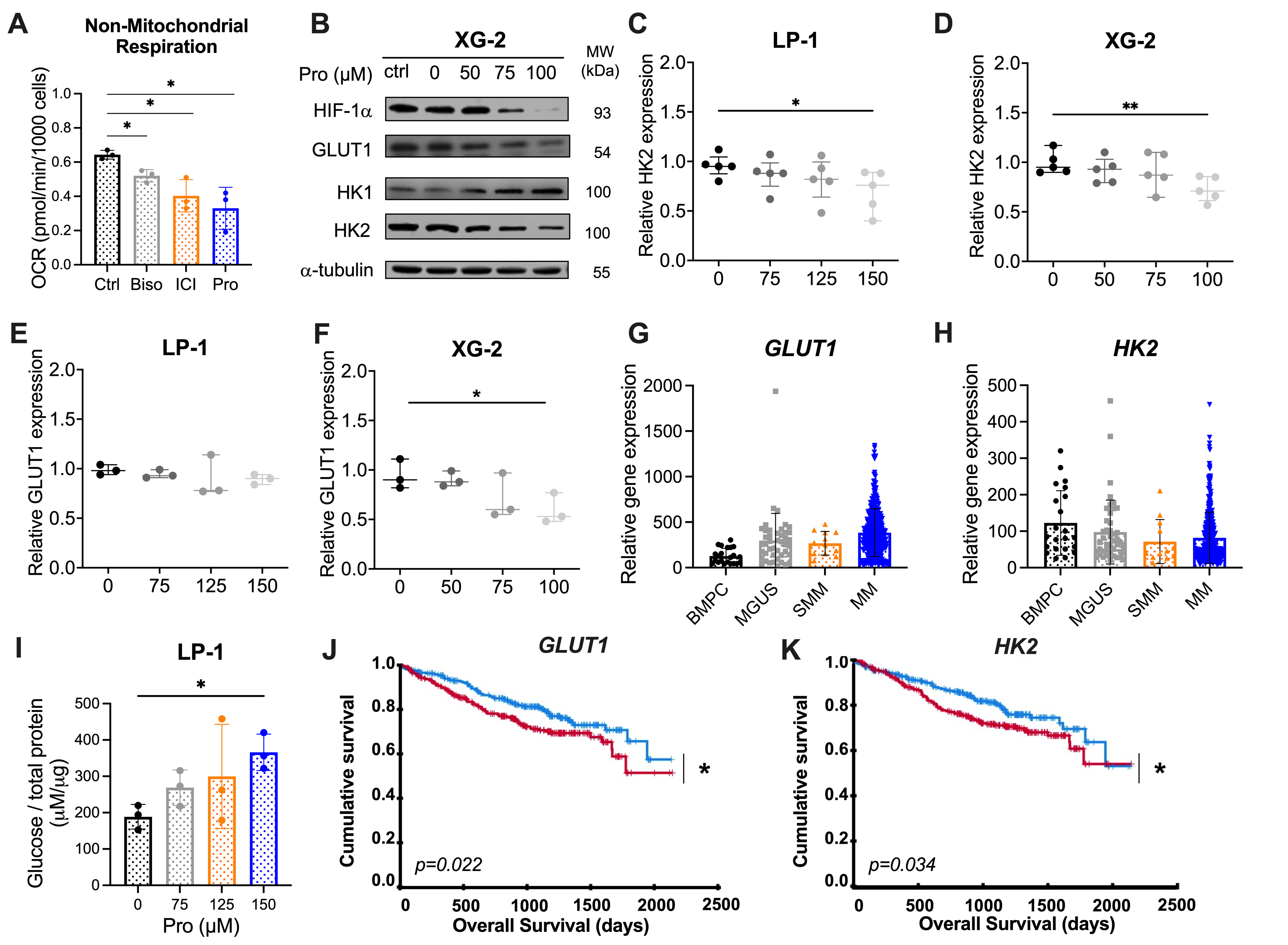


**Figure S5. Quantification of metabolic pathways after propranolol treatment and prognostic value of metabolic markers in multiple myeloma.**

(A) Non-mitochondrial respiration following 24 h β-blocker treatment, measured using a Seahorse analyzer. (B) Western blot analysis of metabolic markers involved in the glycolytic pathway HIF-1α, GLUT1, HK1, and HK2 expression in XG-2 cells following 24 h exposure to propranolol. All markers were analyzed for five independent experiments; one experiment is shown. $\alpha$-Tubulin was used as a loading control. (C–F) Quantification of western blot analysis of HK2 (C, D) and GLUT1 (E, F) in LP-1 and XG-2 cells using ImageJ software. Statistical analysis was performed using a one-sided Mann–Whitney *U*-test, with *p* < 0.05 (*) and *p* < 0.01 (**) considered statistically significant (*n* = 5 for HK2 and *n* = 3 for GLUT1). (G, H) Gene expression of *GLUT1* (G) and *HK2* (H) in healthy BMPCs (*n* = 22), MGUS (*n* = 44), SMM (*n* = 12), and multiple myeloma cells of newly diagnosed patients (*n* = 345) from the TT2 cohort (accession number GSE2658). (I) Glucose levels measured using an Amplex Red glucose/glucose oxidase assay kit. Supernatant of LP-1 cells was collected after 6 h propranolol treatment. Mann–Whitney *U*-test, with *p* < 0.05 (*) considered statistically significant (*n* = 3). (J) The prognostic value of *GLUT1* gene expression in newly diagnosed multiple myeloma patients from the MMRF CoMMpass study. A Mantel–Cox test was used to calculate the overall survival curve, with low *GLUT1* expression (blue, *n* = 327) and high *GLUT1* expression (red, *n* = 326). (K) The prognostic value of *HK2* gene expression in newly diagnosed multiple myeloma patients from the MMRF CoMMpass study. A Mantel–Cox test was used to calculate the overall survival curve, with low *HK2* expression (blue, *n* = 326) and high *HK2* expression (red, *n* = 327). **p* < 0.05.


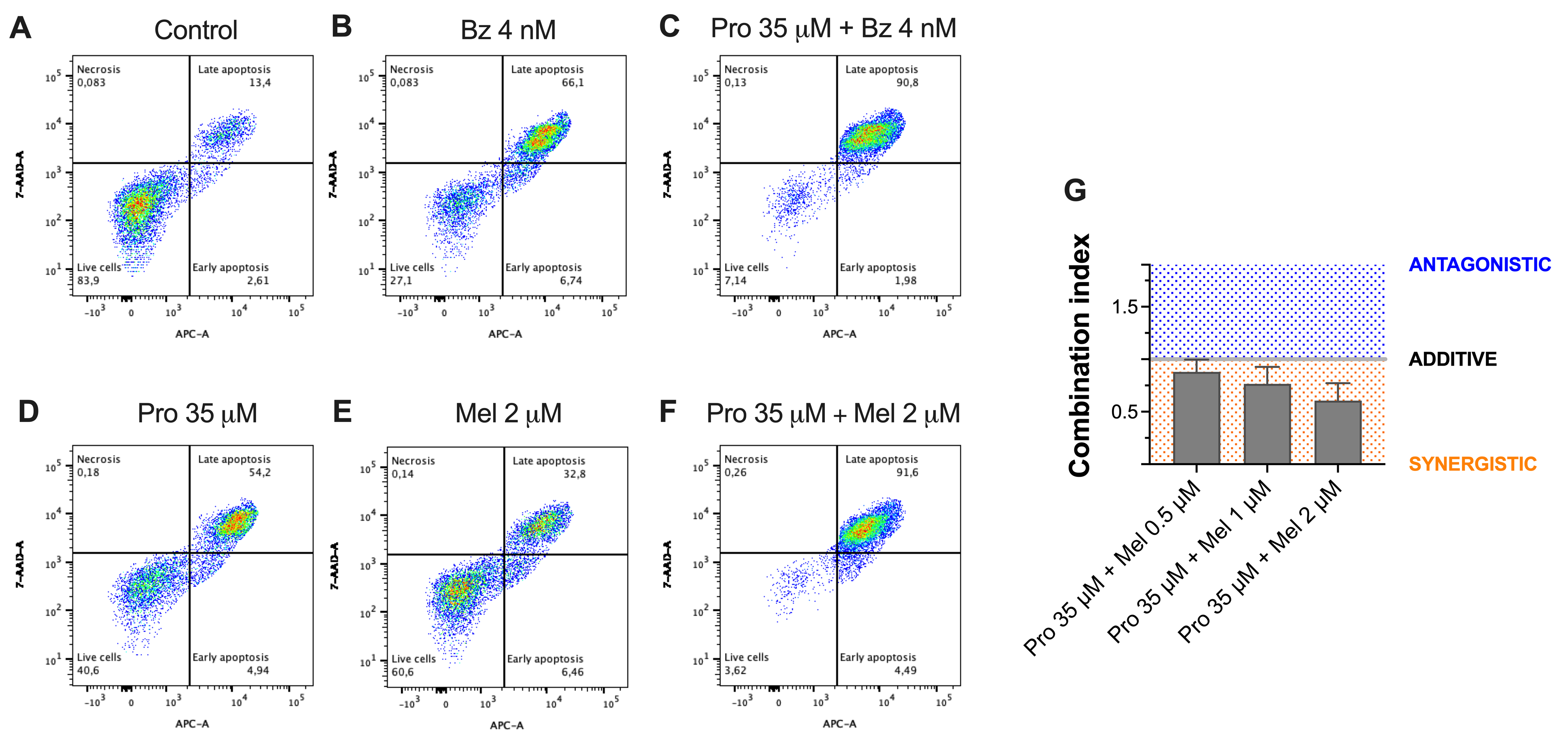


**Figure S6. Propr****anolol in combination with standard-of-care agents increased apoptosis and resulted in synergistic effects.**

(A–F) Apoptosis was analyzed by flow cytometry using an annexin V/7-AAD staining method. Representative scatter plots showing the distribution of annexin V and 7-AAD staining. Cells are classified as ‘live’ (bottom left), ‘early apoptotic’ (bottom right), ‘late apoptotic’ (top right), or ‘necrotic’ (top left). (G) Combination index using the Chou–Talalay method, where CI > 1 indicates antagonism, CI < 1 indicates synergism, and CI = 1 indicates additive effect.
